# A Review of Stellate Ganglion Block as an Adjunctive Treatment Modality

**DOI:** 10.7759/cureus.35174

**Published:** 2023-02-19

**Authors:** Kennedy Kirkpatrick, Mashfee H Khan, Yi Deng, Krishna B Shah

**Affiliations:** 1 Anesthesiology, Baylor College of Medicine, Houston , USA; 2 Anesthesiology, Baylor College of Medicine, Houston, USA; 3 Anesthesiology and Interventional Pain, Baylor College of Medicine, Houston, USA

**Keywords:** adjunctive therapy, ventricular tachycardia, menopause, hot flashes, anosmia, ptsd, peripheral nerve block, long-covid, sgb, stellate ganglion block

## Abstract

Peripheral nerve blocks are becoming increasingly used as adjunctive treatment modalities for a variety of conditions refractory to medical management. Right or left stellate ganglion blocks (SGB) are a specific type of peripheral nerve block that target the sympathetic blockade of neuronal impulses using the injection of local anesthetic and steroids into nerve bundles in the cervical area.

This review article is intended to summarize the common uses of stellate ganglion blocks and explain the procedural technique, which has evolved with technological advances in ultrasonography. The similarities between these disease processes are centered around sympathetic hyperactivity. This sympathetic overdrive state is created by increased levels of nerve growth factor (NGF), which causes a cascade of sympathetic sprouting resulting in increased norepinephrine (NE) systemically. Reversal of this cascade by local anesthetic injection into the stellate ganglion thereby reduces NGF and sympathetic sprouting subsequently lowering overall norepinephrine levels. This is the unifying theory by which SGB is able to provide resolution for the varied clinical conditions described in this article.

This review article discusses the physiology of several conditions where stellate ganglion blocks are being investigated as an adjunct treatment modality, including anosmia, PTSD, long-COVID, chronic fatigue syndrome, menopausal hot flashes, and ventricular tachyarrhythmias. Overall, the current literature supporting the use of stellate ganglion blocks for several esoteric conditions is limited; however, case reports to date have shown promising evidence-based results supporting their use as an adjunctive treatment among patients with refractory symptoms to existing treatment algorithms.

In conclusion, SGB should be considered among patients with refractory symptoms for medical management in the conditions discussed in this article. Further research is needed to delineate which patients will benefit from the use of SGB, the use of subsequent blocks and timelines in between injections, and unilateral versus bilateral blockade.

## Introduction and background

Stellate ganglion blocks (SGB) are being increasingly used for the treatment of many different medical conditions (Table 1).

**Table 1:** Conditions being treated with stellate ganglion block Above is a list of conditions being experimentally treated with SGB that have shown statistically significant relief in their respective disease process in limited case reports to date.

Conditions
Post-traumatic stress disorder
Anosmia
Long-COVID
Chronic fatigue syndrome
Hot flashes, vasomotor symptoms associated with menopause
Ventricular tachyarrhythmias

The effectiveness of SGB in the symptom management of these conditions stems from blocking the sympathetic nervous system (SNS), although the exact mechanism is not well understood [1]. In these patients who are otherwise refractory to conventional management, the efficacy of SGB as an adjunct treatment modality deserves further investigation. This narrative review is intended to summarize the use of SGB in a select group of esoteric conditions, explain the procedural technique and proposed mechanisms of action, address risks and complications, and discuss the current scopes of practice and their efficacy.

## Review

Procedural technique and mechanism of action

Procedural Technique

The stellate ganglion is present in approximately 80% of the population as a fusion of the inferior cervical and first thoracic sympathetic ganglions at the level of the C7 transverse process [2]. In the other 20% of patients, the two ganglia are not fused, so the inferior cervical ganglion is designated as the stellate ganglion. Anatomically, the stellate ganglion is anterolateral to the longus colli muscle, medial to the scalene muscles, anterior to the transverse process and prevertebral fascia, and is separated from the posterior cervical pleura by the suprapleural membrane [2]. Medial to the stellate ganglion are the trachea, esophagus, and vertebral column. Vascular structures that surround the stellate ganglion include the vertebral vessels anterolaterally, the carotid artery anteriorly, the superior intercostal artery laterally, and the costocervical trunk of the subclavian artery inferiorly [2].

Prior to the start of the procedure, care should be taken for proper patient positioning. The patient is placed at a semi-recumbent 30-45-degree angle with a slight extension of the neck. The head is then turned toward the contralateral side. Prior to the use of sonography and fluoroscopy, SBGs were performed utilizing anatomical landmarks by palpating the anterior tubercle of the C6 transverse process (Chassaignac’s tubercle) between the sternocleidomastoid muscle and the trachea, then pushing the carotid artery laterally. The landmark technique is rarely done now due to concerns about both the unpredictability of needle position and the spread of local anesthetic agents [3]. 

**Figure 1:**
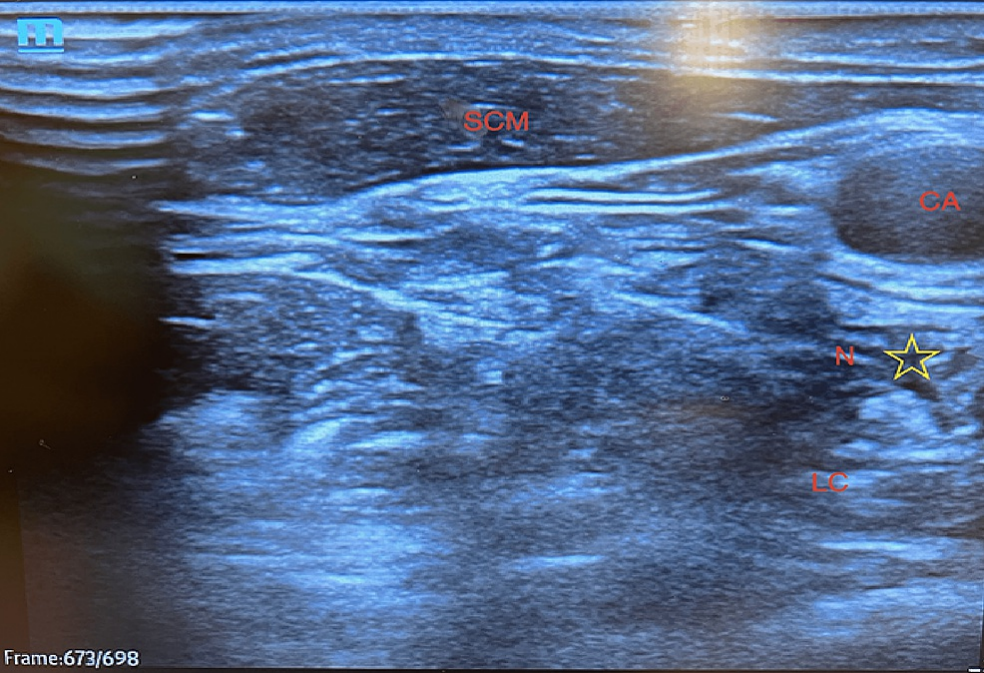
Ultrasound-guided stellate ganglion block at the level of C6 transverse process. SCM: sternocleidomastoid muscle; CA: right carotid artery; LCL longus colli muscle; N: needle; star symbol: sympathetic chain. This figure is an original illustration by the authors of this paper. Ultrasound was obtained during a patient visit by Dr. Krishna Shah. Patient information was deidentified and the screenshot was used for the purposes of this article [4].

Currently, sonographic or fluoroscopic-guided techniques are preferred. During an ultrasound-guided block, a curvilinear transducer is placed at the cricoid cartilage perpendicular to the tracheal axis and moved inferiorly until the thyroid gland is visualized [1]. Next, the transducer is moved laterally to visualize the anterior aspect of the Chassaignac’s tubercle. At this position, multiple structures can be identified, including the carotid artery, internal jugular vein, thyroid gland, trachea, longus colli and capitis muscles, prevertebral fascia, and C6 spinal nerve root (Figure 1).

A color Doppler can be used to detect the position of the vessels. With an in-plane approach, the needle is inserted in a lateral to medial trajectory to reach the prevertebral fascia of the longus colli muscle, located between the carotid artery and Chassaignac’s tubercle [5]. Approximately 5-10 mL of local anesthetic is injected after negative aspiration, with direct visualization of the spread along the paravertebral fascia to the stellate ganglion [1].

If a fluoroscopic approach is used, the patient is placed in a supine position, and the C-arm is used to identify the C6 transverse process. The anteroposterior view is initially obtained, and the C-arm is tilted to line up with the superior aspect of the C6 vertebral body and then rotated obliquely at approximately 25 to 30 degrees to obtain the foraminal view. The target is the junction of the vertebral body and the uncinate process. The needle is then inserted in a lateral to medial trajectory just anterior to the vertebral body or slightly medial to avoid injury to vessels, spinal nerves, and the disc. Approximately 1 mL of contrast is injected first to identify needle position, followed by 1 mL of test-dose local anesthetic, and finally 8-12 mL of therapeutic local anesthetic [1].

Mechanism of Action

**Figure 2:**
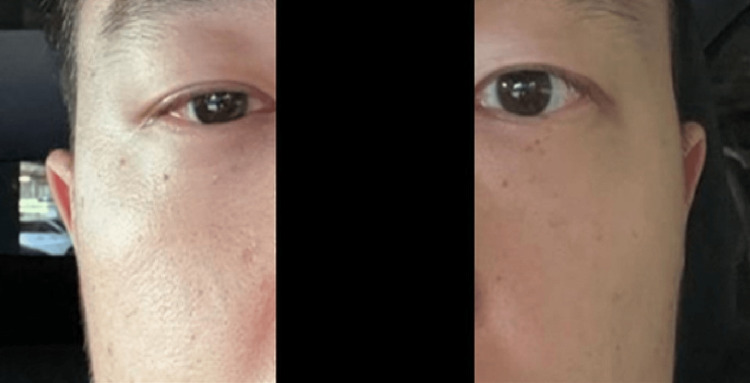
Confirmation of unilateral Horner's syndrome following stellate ganglion block. This figure is an original contribution by the authors of this paper. Photograph was taken of a patient following successful stellate ganglion block for long-COVID demonstrating ipsilateral Horner syndrome [4].

SGB is a selective block of the cervical sympathetic chain that affects the ipsilateral head, neck, upper extremity, and upper thorax. Block efficacy is confirmed by the presence of ipsilateral Horner’s syndrome [ptosis, anhidrosis, miosis, and scleral injection (Figure 2)] [6].

The exact mechanism of SGB is unclear, but most literature suggests it inhibits SNS activity, thus decreasing the chronic stress responses in various disease processes. Earlier literature proposed that the inhibition of SNS-associated peripheral vasodilation resulted in neuronal inhibition at the innervation of the stellate ganglion [7,8]. Newer theories suggest a relationship with nerve growth factor (NGF), which is involved in a variety of signaling events related to acute and chronic stress (Table 2) [9]. Increased NGF has been linked to increases in sympathetic activity and systemic norepinephrine (NE) levels, which can sensitize peripheral nociceptors, causing an increase in perceived pain [10]. SGBs can be useful in blocking afferent nociceptive SNS pathways, thus effectively decreasing NGF levels and resulting in lower sympathetic activity and NE [11].

**Table 2:** Pathophysiology of sympathetic hyperactivity in stressful state compared to post-stellate ganglion block. NGF: nerve growth factor; NE: norepinephrine; PTSD: post-traumatic stress disorder. In chronic stress states, such as PTSD as shown above, levels of NGF are increased as a physiologic response to stress. NGF in turn increases NE. Stress-induced release of epinephrine has been shown to provoke a multitude of anxiety-related behaviors. NGF also increases nerve growth and sprouting that also stimulates NE release. Following SGB, levels of hormones and factors causing sympathetic hyperactivity are decreased resulting in a decrease of complete reversal of anxiety-provoking physiologic changes and restoration of function.

Hormone or factor	Change in each variable during stress
NGF	↑
Sprouting/nerve growth	↑
NE	↑
PTSD/memory dysfunction	↓
Hormone or factor	Change in each variable following SGB
NGF	↓
Sprouting/nerve growth	↓
NE	↓
PTSD/memory function	↑

Risks and complications

The introduction of image-guided SGB significantly lowered complication rates compared to older literature [12-15]. However, it is still associated with the risk of injury to adjacent structures (i.e., cricoid cartilage, carotid artery and other vessels, lung parenchyma, thyroid and parathyroid glands, and esophagus), as well as the risk of intravascular injection and infection [12-13]. SGB at the C6 transverse process is preferred over the C7 due to the lower risk of intra-arterial injection into the vertebral artery. Furthermore, a lateral approach demonstrates fewer complications due to enhanced visualization of anatomic landmarks under fluoroscopy at the lateral tracheal border compared to the anterior approach [1,12,13,16]. Difficulty visualizing this area with an anterior approach results in a higher incidence of esophageal injury or perforation risk [12].

Complications of SGB can be divided broadly into local and systemic. The most common systemic complications of SGB are hoarseness and lightheadedness [17]. Case reports have also shown severe HTN following SGB, likely from spread into the surrounding carotid sheath, resulting in a vagal blockade [18]. Additionally, both short-term and persistent coughs have been noted after SGB, likely from recurrent laryngeal nerve paralysis [19-20]. Local complications most commonly include blood aspiration during injection, intra-procedural bleeding, and hematoma formation. Nevertheless, SGB is a relatively safe procedure, with only five case reports to date documenting delayed hematoma formation requiring tracheostomy and prolonged hospitalization [21-24].

Although image guidance has enhanced visualization of the appropriate targets, anatomic variants or misidentification of relevant structures can result in block failure [14]. Due to the close proximity of multiple head/neck vessels, inadvertent mis-injection can be detrimental [1,12,14,25]. Bhatia et al. demonstrated vertebral artery vulnerability in approximately 12% of cases involving ultrasound-guided SGB [12]. The inferior thyroid artery can also be unpredictable as it ascends anteriorly to the vertebral artery and curves medially behind the carotid sheath [1,14]. Injury to it can cause a retropharyngeal hematoma, leading to serious airway concerns [14]. Lastly, the intra-arterial injection can result in spasms, hematoma, seizure, and other complications, including local anesthetic systemic toxicity (LAST) [26]. Thankfully, LAST is an extremely rare occurrence given the low dose of injectate and is rarely reported in the literature [27].

Esoteric conditions being treated by stellate ganglion block

Post-Traumatic Stress Disorder

PTSD is defined by four clusters of symptoms: intrusive re-experiencing of a traumatic event, avoidance of trauma-related stimuli, negative changes in mood and cognition, and persistent physiological arousal and reactivity. A diagnosis requires symptoms to significantly impair functioning for at least one month. Conventional treatment typically involves psychotherapy and medication, with an overall rate of remission of 30-40% [28], suggesting the need for more innovative approaches.

The putative mechanism of PTSD involves hyperarousal of neuronal processing in the amygdala, anterior cingulate, and orbitofrontal cortex, inducing a hyperactive sympathetic response [29]. This increased cerebral activity makes SGB a plausible treatment option. One proposed mechanism is that SGB reduces NGF, which results in decreased sympathetic nerve sprouting and brain NE levels [29]. This then imparts a sedative effect as measured by decreases in EEG activity on the bispectral index [29], leading to amelioration of hyperarousal symptoms.

The benefits of SGB in PTSD have been suggested in multiple case reports, but a randomized controlled trial was recently performed [30]. In a group of veterans already on stable psychotropic medication, Rae Olmsted et al. found clinically significant improvement eight weeks after the block compared to a sham injection [31]. Participants in the treatment arm received a 7-10 mL injection of 0.5% ropivacaine to their right stellate ganglia, followed by a two-week repeat on the left side. Secondary outcomes showed clinically significant improvements in anxiety, depression, pain scores, and mental and physical functioning [31]. Limitations of this study included narrow patient selection and high PTSD severity at baseline. 

Currently, unilateral right-sided SGB is performed for PTSD given the right hemispheric dominance in controlling survival and stress responses, social and emotional processing, and expression and communication of emotion [32]. Interestingly, in one study, 90% of patients who failed the right-sided block showed significant improvement with a contralateral block [33]. These non-responders may have anatomic variations requiring contralateral intervention, but further research is needed to elucidate the efficacy of left-sided blocks [33].

Thus far, there has been no documented adverse reaction to the addition of SGB to concurrent medical management [29,31,33]. Adverse events are typical for other SBG utilizations, including cough, hoarseness, pain/redness at the injection site, and vasovagal syncope following intravenous catheter insertion [31], although the overall rate of complications is low at 0.017% [15].

In conclusion, while current literature is mostly limited to case reports and potentially handicapped by non-uniform scoring systems, it does seem to support the use of SGB as an adjunct treatment option for PTSD. Typical symptom relief following an injection ranges from three to six months, and repeated blocks may be warranted [33]. Further research is needed to determine the efficacy and timing of repeated blocks as well as unilateral versus bilateral injections. 

Anosmia

Anosmia is defined as the partial or complete loss of smell, and its prevalence is estimated to affect 3-20% of the population [34]. Common causes of anosmia include chronic sinonasal disease, severe head trauma, URI, including COVID-19, and neurodegenerative disease, with the overall risk of anosmia increasing with age [34]. There are few evidence-based treatments, despite the marked negative impacts on quality of life.

Treatment for anosmia is varied and mostly driven by the underlying conditions. The chronic sinonasal disease is treated with steroids with or without antihistamines [35]. In structural cases, surgical intervention may be necessary, although the olfactory outcome is unpredictable. For URI-related anosmia, spontaneous recovery can be observed in 32-66% of patients [36]. Of all current supportive therapies, smell training with patients sniffing different odorants twice a day over a period of four months may be the most effective [37]. Vitamin A nose drops and intranasal citrate have also been tried, but the evidence is mixed.

Alternatively, SGB has been proposed as an emerging treatment modality. Several case reports [38-40] have demonstrated its efficacy in treating specific causes of anosmia. SGB is thought to work similarly by enhancing blood flow during the sympathetic blockade. However, symptomatic relief typically lasts beyond local anesthetic duration and can even be permanent, so one hypothesis suggests that increased neurogenesis due to enhanced circulation is likely responsible.

Given the heightened awareness of COVID-19-induced anosmia (up to 53% of those infected), there have been several case reports recently on the successful utilization of SGB as a cure [39,41]. Chauhan et al. documented a previously healthy female who completely lost olfactory function following a COVID-19 infection and failed multiple conventional treatments [39]. After undergoing an ultrasound-guided SGB, she had a partial return of smell within 24 hours and a complete resolution of anosmia after a contralateral injection 72 hours later. Another case series by Liu et al. highlighted two patients who underwent SGB following COVID-19-induced anosmia [41]. Both patients experienced improved symptoms following the initial block, with complete resolution after the contralateral injection. Symptom relief persisted at the two-month follow-up.

While the current data are limited, SGB seems to be a viable option to attempt refractory anosmia. Although some may criticize the subjective scoring criteria used in many studies, Lee et al. demonstrated statistically significant improvement in both the butanol threshold test and odor identification test following SGB for URI-induced anosmia [40]. Further research is needed to provide guidance on the type of anosmia indicated, timing and laterality, and objective outcome assessment after SGB in this setting.

Long-COVID and Chronic Fatigue Syndrome

CFS is a poorly understood illness defined by severe fatigue lasting longer than six months and at least four of the following symptoms: post-exertional malaise, unrefreshing sleep, impaired memory or concentration, myalgia, polyarthralgia, sore throat, tender lymph nodes, and new-onset headaches [42]. It is a diagnosis of exclusion and is often accompanied by mood disturbances and chronic pain. The pathogenesis is likely multifactorial and hypothesized to involve postinfectious viral etiology, genetic susceptibility, alterations in metabolism, and immune response [42]. Graded exercise therapy was advocated for a long time but has been repudiated in recent years [42]. There is a large need for additional research into this often debilitating disease. More recently, the long-term sequelae of COVID-19 infection, now termed "Long-COVID," was recognized to share overlapping symptomatology with CFS, including fatigue, brain fog, and mental health complications [43]. Long-term COVID is thought to be driven in part by acute tissue injury and hyperinflammation following the viral infection, resulting in vasomotor dysfunction and sympathetic nervous system overdrive [41,44]. As with CFS, there is currently no consensus guideline for long-COVID treatment.

Recent case reports have demonstrated that by targeting the overactive sympathetic activity, SGB can provide symptom relief in long-COVID [41,45]. Liu and Duricka described three patients with prolonged fatigue refractory to treatment and rest after COVID-19 infection [41]. After performing the SGB and then a contralateral injection 24-72 hours later, all three patients experienced sustained symptomatic relief at a 60-day follow-up [41]. Vinyes et al. similarly described a case using 0.5% procaine for both SGB and sphenopalatine ganglion block three times over three months in a long-COVID patient with complete resolution of fatigue [46]. The reason for fatigue recovery is not well understood in this setting. One hypothesis is that COVID-19 is known to damage olfactory neurons, leading to increased CSF outflow resistance and the accumulation of toxic metabolites in the central nervous system (CNS) [46]. In addition to tempering the sympathetic overdrive as aforementioned, SGB by increasing cerebral blood flow may provide additional clearance of these toxic metabolites hence affecting fatigue symptoms.

To date, there has been little research done on the use of local anesthetic injections for CFS. One double-blind, placebo-controlled study had CFS patients undergo lidocaine injections into the trapezius and gluteal muscles versus saline [47]. There was a significant 38% reduction in fatigue scores in the treatment group. The exact mechanism of action is not known. Patients with CFS are thought to have sensitized fatigue pathways, altering peripheral signaling to the CNS [46]. By blocking these peripheral impulses using local anesthetics, central sensitization, and impulses to the CNS may be suppressed, resulting in decreased fatigue levels.

While there is currently no study involving SGB for CFS, given the benefits described for long-COVID fatigue, it is reasonable to further explore this as a potential treatment option for CFS. Further research is needed to elucidate the types of patients that may benefit, the type and dose of local anesthetics, and the frequency and laterality of injections.

Hot Flashes and Vasomotor Symptoms

Hot flashes, also known as vasomotor symptoms (VMS), are one of the most common and distressing symptoms of menopause, experienced by over 75% of menopausal women [48]. Additionally, between 60-80% of patients treated with chemotherapy for breast cancer also suffer from hot flashes [49]. Hot flashes are characterized by recurrent periods of flushing, sweating, and intense heat that begins on the face and upper chest, and can be associated with anxiety, palpitations and red blotching of the skin. Hot flashes are thought to result from a decrease in estrogen levels, however the pathophysiology is not completely understood. One theory postulates that estrogen withdrawal leads to a decrease in endorphin and catechol estrogen levels, which then leads to increased NE and serotonin release [50]. This in turn lowers the set point in the thermoregulatory nucleus and results in vasodilatation and perspiration. NE is thought to be primarily responsible for lowering this set point, with studies showing that plasma levels of NE metabolites are increased both before and after hot flashes [50].

Hormone replacement therapy (HRT) is the first line treatment for reducing hot flashes, however HRT includes increased risk of venothrombotic disease, stroke, and coronary artery disease [48]. It is also generally discontinued after breast cancer diagnosis due to concerns for increasing risk of relapse. Pharmacological alternatives to HRT include gabapentin, clonidine, and antidepressants such as venlafaxine, however their use can be limited by side effect profiles and inconsistent efficacy across trials.  

SGB has been used with promising effect to reduce the severity and frequency of hot flashes, particularly in women with breast cancer who are otherwise not candidates for HRT or refractory to conventional treatment. One suggested mechanism is that the lower perimenopausal estrogen level results in increased NGF concentration, which in turn leads to increased NE levels. SGB is known to reduce NGF levels which may reverse this process and decrease the severity of hot flashes. Multiple case reports and case series have described prolonged symptom relief after SGB [51-53]. A randomized controlled trial in 2014 noted a 52% reduction in the frequency of moderate to severe VMS after SGB compared to 4% reduction in the control group [54]. Another study compared SGB with pregabalin and found a significant improvement in both treatment arms with no adverse events reported in either group [54]. A 2018 trial by Rahimzadeh et al compared SGB with paroxetine and found that both resulted in a significant decrease in hot flash symptoms, with fewer side effects in the SGB group [55]. 

While HRT continues to be the first-line option for VMS, in patients with refractory symptoms or contraindications, SGB is a promising alternative. Limitations include potentially higher cost, lack of availability, and more of the procedure [56]. 

Ventricular Tachyarrhythmias

Ventricular tachycardia, ventricular fibrillation, and electrical storm are among the deadliest arrhythmias, responsible for significant morbidity and mortality. These ventricular arrhythmias (VA) are difficult to treat and can be refractory to conventional medical management, including beta-blockade and antiarrhythmic agents. Catheter ablation is usually the definitive therapy, yet some patients may lack clear targets, not respond to ablation, or be too unstable for transport.

Evidence suggests that SGB can decrease myocardial sympathetic tone, which results in improved short-term control of VA burden and a reduced need for defibrillation. Additionally, successful SGB can increase arrhythmia-free days, giving precious time to stabilize the patient and allowing for more definitive therapy.

Most studies are performed using left-sided SGB for VA treatment, as the left stellate ganglion contributes a greater sympathetic tone to the myocardium than the right [57]. A meta-analysis of 22 case reports published between 1974 and 2017 included 35 subjects already on at least 1 antiarrhythmic and found a reduction in VA burden for at least 24 hours after the blockade [58]. More importantly, due to the stabilizing effect of SGB, 23% of these patients were eventually able to undergo surgical sympathectomy, 23% underwent catheter ablation, and 9% received a heart transplant. Fudim et al. reported the use of SGB in 20 patients with refractory VA, with 45% of patients having no recurrence for 48 hours [59]. Furthermore, the efficacy achieved was independent of VA etiology (ischemic vs. non-ischemic cardiomyopathy) and type (monomorphic vs. polymorphic). To date, however, there has been no randomized trial examining SGB use in VA.

Unlike performing SGB for other etiologies, one complication to note here is LAST. Patients with VA are often on lidocaine or procainamide infusions and can be prone to LAST when given additional local anesthetic [57]. For this reason, some clinicians stop these infusions at least two hours prior to performing the block [57].

SGB can be a useful adjunct therapy for patients with refractory VA. It can also be used as a diagnostic test to determine if a patient’s arrhythmia is responsive to sympathectomy [58]. Serial SGBs can be performed until a more definitive therapy, such as catheter ablation or heart transplantation, can be achieved. Further research is needed to establish whether SGB becomes part of the treatment algorithm for VA.

## Conclusions

SGB is an emerging treatment modality for many sympathetically driven processes. These include the esoteric conditions described in this article which may be difficult to manage with conventional treatments but may be amenable to SGBs. While they show promise in current literature, large multicenter randomized controlled trials are needed in the future to further validate the efficacy of SGB in these settings. Additional research is also needed to elucidate the timing, laterality, and repetition of blocks for these conditions. 
